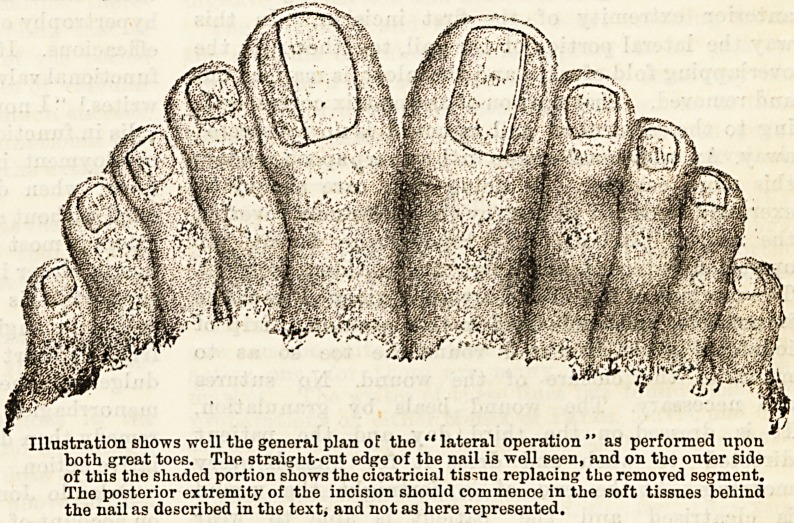# Ingrowing Toenail and Its Radical Cure

**Published:** 1894-10-27

**Authors:** W. Ernest Miles

**Affiliations:** House Surgeon to the Metropolitan Hospital


### Medical Progress and Hospital Clinics.

[*The Editor will be glad to receive offers of co-operation and contributions from members of the profession. All letters should be addressed to* The Editor, The Lodge, Porchester Square, London, W.]

### INGROWING TOE-NAIL AND ITS RADICAL CURE.

By W. Ernest Miles, F.R.C.S.Eng., House Surgeon to the Metropolitan Hospital.

The term ingrowing of the toe-nail is a misleading one, since it implies that the nail, from a faulty direction in its growth, buries itself into the soft tissues of the toe. Authorities are now agreed that such a faulty growth of the nail does not take place, and that the condition is brought about by other causes. Nevertheless, it is convenient to retain the term for want of a better. The affection is a common one, and the surgeon, whether he is engaged in hospital or in private practice, will be called upon to treat it, and, still more, will be expected to effect a cure. It often happens that slight ailments, as they are termed, are less amenable to treatment than more important ones, and ingrowing of the toe-nail is no exception. It is the purpose of this paper to show that the disease may be successfully dealt with by studying the causes that produce it, and by placing the treatment upon a rational and sound basis. The great toe is the one that is most commonly affected, but the others are by no means exempt. I cannot recollect ever having seen a case where the nail of the little toe was at fault.

**Causes.** — Most authorities are of opinion that the condition is produced by the undue pressure of tightly fitting and badly constructed boots. The fashionable boot with its pointed toe, and more especially the high-heeled shoe worn by most women, are undoubtedly potent causes. In the one case the toes are cramped up into a smaller space than is natural to them, and in the other, the whole weight of the body is practically thrown upon the anterior part of the foot. The effect of this is that the soft tissues are made to press upon and overlap the lateral margin of the nail, a small ulcer forming at the point of greatest pressure. The great toe being more prominent than the rest, in addition to carrying the largest and most unyielding nail, suffers more often. That this condition is produced by pressure and not by a faulty direction in the growth of the nail, is evidenced by the fact that the growth of a nail takes place solely from behind forwards and never laterally, and also by the fact that a similar condition is never met with in the hands. Quite recently, however, some French authorities have ascribed the disease to other causes. M. Binaud has described the disease as occurring in bed-ridden tuberculous patients, and Poucet, of Lyons, in persons of lymphatic temperament. In these instances, however, M. Regnault* attributes the morbid condition to injury, and points out that this, together with want of personal cleanliness, favours the growth of ordinary pyogenic microbes in the abrasion along the margin of the nail.

**Local Condition.**—If a toe, the subject of ingrowing toe-nail be examined, and the overlapping fold of skin be drawn aside, a small ulcer will invariably be found, situated along the lateral margin of the nail, at the bottom of the groove between the nail and the adjacent soft tissues. The surface of this ulcer is covered by exuberant granulations, and is exquisitely tender to the touch. There is also a small amount of fœtid discharge. Cutting the nail short only aggravates the condition, and in time the patient is unable to bear the slightest pressure on the toe. Thus it happens that the patient is deprived of exercise from the intense suffering he experiences while wearing a boot or shoe, and in time his general health is impaired, and his life made a burden to him. Cases such as these frequently apply to the surgeon for relief, and since the sufferers are unwilling to dispense with the fashionable boot, a radical operation becomes necessary.

Illustration shows well the general plan of the "lateral operation" as performed upon both great toes. The straight-cut edge of the nail is well seen, and on the outer side of this the shaded portion shows the cicatricial tissue replacing the removed segment. The posterior extremity of the incision should commence in the soft tissues behind the nail as described in the text, and not as here represented.

**Treatment.**—Various methods of procedure have from time to time been adopted for the relief of this condition, such as paring away the nail on the side affected; filing the nail along the middle line until it is as thin as parchment, so that it may be more yielding; avulsion of the whole nail; and, lastly, the removal of the whole matrix as well as the nail. The last of these certainly cures the disease, but it has this objection, and one that is strongly urged by patients themselves, namely, that the scar tissue replacing the lost nail constitutes a deformity. In regard to the others above-mentioned, my experience is that they afford but temporary relief, and that sooner or later the patient again presents himself for treatment. The operation that I now always perform, and which I have found to yield most satisfactory results is one of which I think I can justly claim to be the

* Association Française pour l'Avancement des Sciences, 1894.

originator since I have never seen it described by any one else. It is performed as follows:—

**Operation.**—The patient having been anæsthetised with ether or chloroform, or local anæsthesia having been produced by means of the ether spray, the point of the knife is introduced into the soft tissues behind the nail, at a point midway between the base of the terminal phalanx and the crescentic fold of skin at the root of the nail, along a line drawn longitudinally through the nail, midway between its middle line and its lateral border. From this point an incision is carried forwards, dividing the nail and its underlying matrix in its whole length, terminating in the soft tissues at the extremity of the toe. It is necessary to commence the incision at the point indicated so as to be behind the posterior extremity of the root of the nail, which extends beneath the skin for some little distance. It is well also not to make the incision further back than this for fear of wounding the synovial sac of the last interphalangeal joint, which overlaps to some extent the base of the terminal phalanx. From the posterior extremity of this incision, a second curved incision is made in the soft tissues, skirting the lateral margin of the nail and ending in the anterior extremity of the first incision. In this way the lateral portion of the nail, together with the overlapping fold of skin and the ulcer, is mapped out and removed. That portion of the matrix corresponding to the segment of nail removed is now dissected away. An additional scrape with a sharp spoon renders this more certain. In doing this, care should be exercised so as not to destroy the periosteum covering the ungual phalanx. The hæmorrhage during the operation is trivial, and a ligature is seldom required. The margins of the wound are approximated, so far as is possible, and while held in this position a strip of iodoform gauze is wound round the toe so as to maintain the closure of the wound. No sutures are necessary. The wound heals by granulation. It is dressed on the third day, and the patient directed to wash and dress it for himself every morning. At the end of a fortnight the wound is cicatrised, and the patient is able to wear his boot with comfort. There is no necessity for keeping the patient in bed during any part of the after-treatment, since he is quite able to walk about in a slipper of which the part covering the toe has been cut away.

The accompanying illustration has been made from a photograph which was taken from a patient, upon both of whose great toes I operated at the same time. At the end of three weeks he was able to accompany his volunteer battalion to the Aldershot manœuvres, and marched on an average twenty miles per day without pain or discomfort. The photograph was taken on his return. The rationale of the operation, which might conveniently be termed the "lateral operation," is, that the portion of the nail that gives rise to the trouble is removed, together with its underlying matrix, thereby ensuring no regeneration; that by commencing the incisions in the soft tissues behind the nail, that portion of the matrix that is situate beneath the skin is also removed; and that the portion of the nail left *in situ* is narrower than the toe, and therefore is not likely again to be encroached upon.

## Figures and Tables

**Figure f1:**